# Flexitarian Diet and Weight Control: Healthy or Risky Eating Behavior?

**DOI:** 10.3389/fnut.2018.00059

**Published:** 2018-07-10

**Authors:** Catherine A. Forestell

**Affiliations:** Department of Psychological Sciences, The College of William and Mary, Williamsburg, VA, United States

**Keywords:** flexitarian, weight control, restrained eating, health motivations, semi-vegetarian

## Abstract

A carefully planned vegetarian diet meets nutrition recommendations by providing essential nutrients and lowering levels of saturated fat and cholesterol. Because balanced diets that limit or exclude meat tend to be lower in calories than omnivorous diets, it has been suggested that vegetarian eating patterns may be motivated by weight control. This view has been supported by findings demonstrating that vegetarians have a higher rate of disordered and restrained eating than non-vegetarians. Other findings suggest that weight control is a primary reason identified by adolescents and young adults for eliminating items such as meat and other animal products from their diet. Thus, it has been suggested that vegetarianism may provide a socially acceptable means to control body weight. However, this may be an over-generalization. Vegetarians are a heterogeneous group of individuals with radically different eating habits. Moreover, they are often compared to omnivores who eat meat on a regular basis. These omnivorous eating habits do not represent a growing subset of the population, many of whom are adopting a flexitarian diet that involves only the occasional consumption of meat. The goal of the current paper will be to demonstrate that semi-vegetarians and flexitarians are categorically different from vegans, lacto-ovo-vegetarians, and omnivores and describe the motivations as well as the positive and negative health implications that are associated with dietary patterns that limit the intake of meat. It is important for us to understand the motivations and behaviors that are characteristic of flexitarians in order to develop effective evidence-based strategies to address unhealthy eating behaviors.

## Introduction

The consumption of meat is associated with affluence and wealth and is culturally embedded within the society of most developed countries. It is considered by many to be the central element of a meal and a necessary component of a healthful diet (1). Although meat products provide a wide range of nutrients, such as proteins, fat, and vitamins, recent recommendations suggest that diets that are high in vegetables, fruits, whole grains, and lower in red and processed meat have the most desirable health outcomes (2). The goals of the present paper are to review the motivations as well as the positive and negative health implications that are associated with dietary patterns that limit the intake of meat.

The degree to which individuals choose to consume or avoid animal products varies along a continuum (3–5). Generally those who avoid consuming food that contains animal flesh are referred to as vegetarians. However, vegetarians vary widely in their dietary habits. For example, while vegans avoid all animal products and consume only foods derived from plants, lacto-ovo vegetarians consume dairy and eggs, and semi-vegetarians limit certain types of flesh, such as red meat, fish, or poultry. Like vegetarians, the frequency with which omnivores consume meat can vary along a continuum, with a growing proportion choosing limit their meat consumption (6). Although some use the term semi-vegetarian to define those who eat a mostly vegetarian diet but occasionally eat meat (7), in this paper these individuals will be referred to as flexitarians.

## Motivations to reduce meat intake

There are a variety of dietary motivations among those who can afford to buy meat, but choose not to eat meat. For many throughout the world, this dietary practice reflects their religious persuasion. Many religious faiths have dietary customs or practices that involve limiting certain meat products from the diet during specific holidays (e.g., Roman Catholicism) or throughout the year (e.g., Buddhism, Judaism, Islam, Seventh Day Adventist) (8).

In addition to religion, there is a range of secular motivations for avoiding the consumption of meat (9). These motivations can reflect sensory reactions to meat, such as feelings of disgust or repugnance (10–12), beliefs about the ethical treatment of animals (13, 14), and concerns about personal health and planetary well-being (15–18). Those who have strong religious or ethical reasons for avoiding animal products tend to adopt more restrictive forms of vegetarianism (19–21) relative to those who reduce their meat intake for health or environmental reasons. Typically flexitarians fall between vegetarians and full-time meat eaters in the degree to which they endorse health attitudes (22), and issues concerning human and animal welfare. Thus, it is important to consider flexitarians as a separate group of consumers; they are as different from full-time meat eaters as they are different from vegetarians in their moral (23) and health-related attitudes (16) and behaviors.

Flexitarianism has been presented as a dietary strategy to address concerns about the health and environmental consequences of eating meat (24). Although meat is often associated with pleasure and personal and social values (25), there is evidence to suggest that the excessive production and consumption of meat may be adversely affecting the general well-being of the human population and environment. Increased awareness of the indirect effects of meat production on health, such as widespread use of antibiotics and the propagation of pathogens and greenhouse gas emissions (26, 27) has caused concern about the sustainability of meat consumption. There is also increased awareness that meat may not confer the health benefits once believed. Research has shown that cardiovascular diseases, such as cancer, type 2 diabetes, and obesity are linked to meat intake (28–30). Indeed, for flexitarians the option of cutting back on meat, rather than abstaining completely, is a practical compromise that could have meaningful implications for environmental sustainability and personal health.

## Reduced meat intake and weight

Evidence suggests that dietary patterns that reduce meat consumption may be protective in our current obesity-promoting environment. This has been demonstrated in the Adventist Health Study-2 cohort, a cross-sectional longitudinal study that followed 97,000 Adventist church members beginning in 2002 (31, 32). The Adventist Church prohibits the consumption of biblically unclean foods, such as pork and shellfish, and recommends consumption of fruits, vegetables, wholegrain cereals, legumes and nuts, and avoidance of meats (33). When participants' dietary habits were categorized according to the frequency with which they consumed animal products, results showed that participants' body mass index (BMI) differed according to the degree to which they limited meat (*p* < 0.0001). Vegans were the only group who reported a healthy BMI of less than 25 kg/m^2^ and the BMI of the other dietary groups increased incrementally with reported increases in the frequency of meat and animal product intake (31, 32). The positive relationship between the frequency of meat consumption and BMI may occur for several reasons. First, people who reduce meat from their diets typically engage in other non-dietary lifestyle habits that promote weight loss and health (34, 35). Second, those who reduce intake of meat in their diet consume more plant-based foods compared to those who do not reduce meat. Finally, because animal products tend to be high in saturated fat, their intake may cause weight gain.

## Can reduced meat intake serve as a mask for disordered eating?

Given their perceived effects on weight loss, dietary patterns that involve reduced meat intake may be employed as a socially accepted approach to engage in maladaptive weight control strategies (36). As summarized in Table 1, a number of studies have investigated the relationship between vegetarianism (broadly defined) and its relationship to disordered eating. To do this, some researchers have examined the prevalence of vegetarianism in samples of individuals with eating disorders. These studies have revealed that approximately a quarter to half of those suffering from eating disorders such as anorexia nervosa and bulimia nervosa currently identify (or previously identified) as vegetarians (37–40). Other studies, have assessed the prevalence of eating disorders in samples of vegetarians and have reported that approximately a third to half of vegetarians are at risk for eating disorders (41–43). In addition, studies have compared vegetarians to non-vegetarians on a number of variables related to weight control and dieting. While some researchers have concluded that vegetarianism is associated with dieting and weight control (43–47), others have failed to find differences between vegetarians and non-vegetarians on dieting and weight control measures (48), or have found the opposite effect (5).

**Table 1:** Summary of studies that have assessed the relationship between the degree to which people reduce their meat consumption and restrained eating and/or eating disorders.

**Reference; Country**	**Participants; Sample composition^a^**	**Outcome variable(s)^b^; Measurement scale**	**Findings**
(5), Canada	45 women (age: 20–40 years); 23 vegetarians and 22 omnivores (consuming three or more servings of meat per week)	Dietary restraint; Three Factor Eating Questionnaire (TFEQ)	Dietary restraint was higher in omnivores than vegetarians.
(36); UK	131 undergraduate women; 45 current and former vegetarians^c^ and 86 omnivores	Dietary restraint; Dutch Eating Behavior Questionnaire (DEBQ)	Dietary restraint was higher among vegetarians than among meat eaters. No differences between groups was found in proportion who reported that they were dieting.
(37); USA	160 women (age: >16 years); 93 with a history of eating disorders, 67 with no prior history of eating disorders	Vegetarianism; Self-reported current and lifetime vegetarianism^c^	More individuals with a history of an eating disorder reported ever having been a vegetarian, as well as currently being vegetarian, compared to individuals with no eating disorder history.
(38), UK	180 men and women with anorexia nervosa	Vegetarianism; retrospective analyses of case notes. Vegetarianism was categorized as “absent,” “occasional,” “usual,” and “severe”	82 had been vegetarian as a pervasive feature at some stage of their illness and of these 77 patients were vegetarian (29 “usual,” 48 “severe”) at the time of first attendance at the clinic. The remaining 98 patients were omnivores (88 “absent,” 10 “occasional”).
(39); Australia	116 patients with anorexia nervosa.	Vegetarianism; retrospective analyses of case notes.	Sixty-three patients were semi-vegetarian. In four of these patients, meat avoidance predated the onset of their anorexia nervosa.
(40); USA	278 women; 69 with a diagnosed eating disorder (clinical), 136 who endorsed recent eating pathology (subclinical), 73 who denied any eating pathology (non-clinical)	Vegetarianism: Self-reported current and lifetime vegetarianism and assessed consumption of various food items.	The prevalence of lifetime vegetarianism was significantly higher in the clinical group compared to the subclinical group, which in turn was significantly higher than the nonclinical group. Those in the clinical group were more likely than those in both other groups to self-identify as current vegetarians.
(41); Turkey	1205 undergraduate men and women; 31 vegetarians^c^ and 1174 omnivores	Disordered Eating; Eating Attitudes Test (EAT).	The mean EAT-26 score of the vegetarian participants was higher than that of the omnivorous participants of both genders.
(42); USA	143 undergraduate women; 30 vegetarians^c^ and 113 omnivores	Disordered Eating; EAT.	The median EAT score of the vegetarians was significantly higher than that of the non-vegetarians. A greater proportion of vegetarians scored higher than 30 on the EAT compared with the omnivores.
(43) Canada	596 undergraduate women; 47 vegetarians and 549 omnivores	Dietary restraint; TFEQ Disordered eating; self-report of diagnosis	Those who had higher restraint scores were more likely to be vegetarian. Relative to omnivores, a higher percentage vegetarian participants reported an eating disorder diagnosis.
(44); USA	321 male and female adolescents (age: 12–20 years); 107 vegetarians^c^ and 214 omnivores	Disordered eating; questions about frequency of dieting, and whether they engaged in binge eating, self-induced vomiting, and laxative use	A higher percentage of vegetarian adolescents reported engaging in frequent dieting, binge eating, self-induced vomiting, and laxative use compared to omnivores.
(45); USA	2,516 men and women (age: 15–23 years); 2,112 omnivores, 108 current vegetarians^c^, 268 former vegetarians.	Binge eating; 2 questions that assessed loss of control over eating.	Current vegetarians reported engaging in binge eating with loss of control when compared to never vegetarians.
(46); Canada	224 men and women (age: 15 −45 years); 70 vegetarians, 49 semi-vegetarians, 105 omnivores	Dietary restraint; TFEQ Feminism was measured as a moderator variable.	Dietary restraint was positively correlated with the degree to which men and feminist women reduced their meat intake.
(47); Australia	1070 female adolescents (mean age: 16 years); 245 vegetarians^c^, 825 omnivores	Dietary restraint; TFEQ	Dietary restraint was higher in vegetarians than in omnivores.
(48); USA	256 undergraduate women; 52 vegetarians^c^ and 204 omnivores	Dietary restraint; DEBQ and TFEQ Disordered eating; Eating Disorder Inventory (EDI-II) and EAT	Dietary restraint and disordered eating behavior did not differ between vegetarians and omnivores.
(49); USA	240 undergraduate women; 55 vegetarians, 28 pesco-vegetarians, 29 semi-vegetarians, 37 flexitarians, 91 omnivores	Dietary restraint; TFEQ Disordered eating; EAT	Dietary restraint scores were significantly higher in semi-vegetarians and flexitarians relative to omnivores. However, restraint scores of vegetarians and pesco-vegetarians did not differ from omnivores. There were no significant between-group differences in disordered eating.
(50); USA	Experiment 1: undergraduate and community men and women; 35 vegans, 111 vegetarians, 75 semi-vegetarians, and 265 omnivores Experiment 2: undergraduate women; 44 semi-vegetarians and 74 omnivores	Dietary restraint; DEBQ Disordered eating; EAT-26 Dietary restraint; Restraint Scale (RS) Disordered eating; Eating Disorder Examination Questionnaire (EDE-Q)	Dietary restraint significantly differed across groups; vegans had significantly lower levels of restraint than semi-vegetarians, and semi-vegetarians had higher levels of restraint than omnivores. Semi-vegetarians had marginally higher scores than the other groups on the EAT-26. Dietary restraint and eating concerns (subscale of EDE-Q) were higher in semi-vegetarians than omnivores. There were no significant differences on the weight concern, shape concern and restraint subscales of the EDE-Q.
(51); USA	90 undergraduate, faculty, and community women (age: 18–57 years); 20 vegetarians, 16 semi-vegetarians, 54 omnivores	Dietary restraint; TFEQ Weight control motivation; rank-order food motivation questionnaire	Dietary restraint was significantly higher in semi-vegetarians and non-vegetarians compared to vegetarians; weight-motivated semi-vegetarians reported higher levels of dietary restraint than did weight-motivated vegetarians

a *Sample sizes and groups are reported based on those included in the primary analyses conducted in each study*.

b *Although many studies measured multiple outcome variables, only restrained eating, disordered eating, and vegetarianism are reviewed*.

c *In addition to those who limit all meat and fish from their diet, this group consisted of semi-vegetarians who consumed poultry and/or fish*.

There are a number of limitations that have plagued research in this area. First, the inconsistent findings may be a result of the variation in the composition of vegetarian samples. Much of the research published to date has not differentiated subgroups of vegetarians. Rather this work has either focused only on vegans and lacto-vegetarians [e.g., 5], or has compared a heterogeneous sample of vegetarian and vegetarian-oriented individuals as a whole to omnivores (36, 37, 41, 42, 44, 45, 47, 48). This is often done because of the difficulty in recruiting sufficient numbers of vegetarian subgroups to have enough power for meaningful statistical analysis. Studies that fail to distinguish between different subgroups of vegetarians can be misleading because these subgroups can vary widely in their beliefs and in their dietary motivations. Even within each subgroup, there are different motives that direct dietary habits (22). These issues are further complicated by the common reliance on self-report, in which participants are asked to identify themselves as vegetarians, often without the benefit of an operational definition. However, if participants are not asked to report the frequency of their meat intake, it is difficult to determine whether their identification as a vegetarian actually reflects their dietary habits.

Second, previous research has either included a limited sample of omnivores, including only those who reported eating red meat at least three times a week [e.g., (5)], or has not asked omnivores to indicate how frequently they eat meat (36, 41–47). Because previous studies have ignored flexitarians, or included them with other omnivores who do not restrict their meat intake, it is unclear what factors motivate their food intake.

## Flexitarianism and weight control

More recent research suggests that maladaptive eating habits, such as restrained eating may be more common in flexitarians (49) compared to vegetarians who restrict all forms of flesh from their diet. Restrained eating reflects a struggle to maintain control over food intake and weight (52, 53), which is often interrupted with episodes of overeating (54, 55). As a result, restraint is not associated with a reduction in overall caloric intake. Although restrained eating differs from disordered eating, it is often used as a marker for disordered eating and is believed to be a risk factor for the development of an eating disorder (56, 57).

In the only study that has investigated flexitarians' restrained eating and disordered eating patterns, Forestell et al. (49) sought to determine whether restrained eating behaviors of subgroups of vegetarians differed from those of flexitarians and omnivores. They asked participants to indicate whether they adhered to a vegan; lacto-vegetarian; ovo-vegetarian; pesco-vegetarian; semi-vegetarian; flexitarian; or an omnivorous diet, providing clear operational definitions of each dietary pattern. To verify these self-reports, they interviewed participants regarding the frequency with which they ate a variety of foods including various meat products over the previous year. In this manner, they were able to prevent individuals who misrepresented their diets from biasing the results. In addition, they assessed participants' restrained eating behavior using the Restrained Eating subscale of the Three Factor Eating Questionnaire (TFEQ) (58).

Findings from this cross-sectional study suggest that the restrained eating patterns of vegans and lacto-ovo-vegetarians (combined) and pesco-vegetarians did not differ from those of omnivores. However, semi-vegetarians and flexitarians were significantly more restrained than the other groups (43). This finding is consistent with other research that found that semi-vegetarians were significantly more restrained than lacto-ovo-vegetarians and vegans (50, 51).

Although the sample recruited by Forestell and colleagues (43) was large enough to compare vegetarian and omnivorous subgroups' restrained eating behaviors, it was limited in that it consisted of young American college women from a narrow demographic background. Despite this limitation, it appears that vegetarianism is not necessarily a primary factor in the etiology of disordered eating. Rather less extreme forms of meat restriction (i.e., semi-vegetarian and flexitarian dietary patterns) appear to be associated with restrained eating. As discussed above, flexitarians and semi-vegetarians are likely to identify health and environmental concerns as a reason for reducing their meat intake. It is possible that for those who identify health as a motivator, this drive primarily embodies concerns about weight management and reduction. This is consistent with studies showing that those who endorse reasons other than weight concerns for meat restriction, have significantly lower levels of dietary restraint than those who endorse weight concerns (51).

## Conclusions

Whether flexitarians and semi-vegetarians are more vulnerable to engaging in maladaptive eating than those who engage in more extreme forms of meat restriction across all ages and genders remains to be seen. Future research should replicate and extend findings reported by Forestell and colleagues by investigating how factors, such as personality variables, motivations, and lifestyle habits, may predict unhealthy approaches to eating in flexitarians over the long term. For example, research shows that there are differences in levels of depression between semi-vegetarians, vegetarians, and omnivores (59). Given that restrained eating is positively related to depression (60), more research is needed to understand how restriction of meat, depressive tendencies, and restrained eating are related.

Those who follow well-planned vegan or vegetarian diets typically consume foods that are low in saturated fat and high in fiber, both of which contribute to weight control. It is important to note however, that unlike many flexitarians, vegan and vegetarians are less motivated by weight control than by other factors such as concern about health, the environment, or dislike of the taste of meat (43, 50). While it is generally believed that flexitarian and semi-vegetarian eating habits may be motivated by concerns about personal and environmental well-being, there is evidence that weight control may also be a concern for some individuals within this group (43, 50). It is important for future research to investigate these individual differences within subgroups. It is also important to consider how motivations for meat restriction change over time, as research suggests that vegetarians' motivations for limiting consumption of meat are not static, but instead change and evolve (15). Once we have a better understanding of the motivational factors related to flexitarianism and whether and in what situations this dietary approach is associated with restrained eating and disordered behaviors, we will be able to develop more tailored approaches for identifying and helping those who mask their maladaptive eating behaviors by reducing their meat consumption.

## Author contributions

The author confirms being the sole contributor of this work and approved it for publication.

### Conflict of interest statement

The author declares that the research was conducted in the absence of any commercial or financial relationships that could be construed as a potential conflict of interest.
